# ADP Induces Blood Glucose Through Direct and Indirect Mechanisms in Promotion of Hepatic Gluconeogenesis by Elevation of NADH

**DOI:** 10.3389/fendo.2021.663530

**Published:** 2021-04-27

**Authors:** Xinyu Cao, Xiaotong Ye, Shuang Zhang, Li Wang, Yanhong Xu, Shiqiao Peng, Yang Zhou, Yue Peng, Junhua Li, Xiaoying Zhang, Xiao Han, Wen-ying Huang, Weiping Jia, Jianping Ye

**Affiliations:** ^1^ Shanghai Diabetes Institute, Shanghai Jiao Tong University Affiliated Sixth People’s Hospital, Shanghai, China; ^2^ National Demonstration Center for Experimental Fisheries Science Education, Shanghai Ocean University, Shanghai, China; ^3^ Core Facility Center of the First Affiliated Hospital of Nanjing Medical University, Nanjing, Jiangsu, China; ^4^ College of Physical Education, Jiangxi Normal University, Nanchang City, China; ^5^ Key Laboratory of Human Functional Genomics of Jiangsu Province, Department of Biochemistry and Molecular Biology, Nanjing Medical University, Nanjing, China

**Keywords:** ADP, mitochondria, NADH, gluconeogenesis, purinergic receptor, P2Y13

## Abstract

Extracellular ADP, a derivative of ATP, interacts with the purinergic receptors in the cell membrane to regulate cellular activities. This signaling pathway remains unknown in the regulation of blood glucose *in vivo*. We investigated the acute activity of ADP in mice through a peritoneal injection. In the lean mice, in response to the ADP treatment, the blood glucose was elevated, and pyruvate tolerance was impaired. Hepatic gluconeogenesis was enhanced with elevated expression of glucogenic genes (*G6pase* and *Pck1*) in the liver. An elevation was observed in NADH, cAMP, AMP, GMP and citrate in the liver tissue in the targeted metabolomics assay. In the primary hepatocytes, ADP activated the cAMP/PKA/CREB signaling pathway, which was blocked by the antagonist (2211) of the ADP receptor P2Y13. In the circulation, gluconeogenic hormones including glucagon and corticosterone were elevated by ADP. Insulin and thyroid hormones (T3 and T4) were not altered in the blood. In the diet-induced obese (DIO) mice, NADH was elevated in the liver tissue to match the hepatic insulin resistance. Insulin resistance was intensified by ADP for further impairment in insulin tolerance. These data suggest that ADP induced the blood glucose through direct and indirect actions in liver. One of the potential pathways involves activation of the P2Y13/cAMP/PKA/CREB signaling pathway in hepatocytes and the indirect pathway may involve induction of the gluconeogenic hormones. NADH is a signal for gluconeogenesis in the liver of both DIO mice and lean mice.

## Introduction

Adenosine triphosphate (ATP) is an important energy molecule in cells, and surplus of intracellular ATP is a risk factor of insulin resistance (1). In the cells, ATP surplus leads to suppression of adenosine 5’-monophosphate (AMP)-activated protein kinase (AMPK) activity (2), which accounts for reduced glucose transporter 4 (Glut4) activity in the insulin signaling pathway for insulin resistance. In an alternative pathway, ATP surplus leads to activation of mammalian target of rapamycin (mTOR) (3), which induces the downstream S6 kinase (S6K) to impair insulin sensitivity by serine phosphorylation of insulin receptor substrate 1 (IRS-1) (4, 5). Metformin inhibits ATP production in mitochondria to induce AMPK and inhibit mTOR, which are associated with improvement of insulin sensitivity and suppression of hepatic gluconeogenesis (6). These studies suggest that intracellular ATP may be a risk factor of insulin resistance. However, the possibility remains to be tested with an accurate quantification of ATP.

ATP may regulate cell metabolism through an action in the extracellular matrix. ATP is secreted or released to the extracellular matrix to act as a signaling molecule. In the physiological conditions, the secretion occurs together with release of hormones, cytokines or neurotransmitters by cells (7). In the pathological conditions, ATP is released upon cell damage by necrosis, hypoxia and infection (8, 9). The activity has been reported in macrophages, β-cells, hepatocytes, neuronal cells and endothelial cells (10–13). ATP concentration may reach 3.5 mmol/L and ADP concentration may reach 5 mmol/L in the insulin-secretory granules of β-cells (14). The ATP level may exceed 25 μmol/L in the extracellular matrix in the pancreatic islet (15). ATP is rapidly degraded by ectonucleotides to generate ADP and AMP in the extracellular space (13). The extracellular ATP activities have been well documented in the fields of immunology/inflammation (7, 16). However, the ADP activity remains largely unknown, especially in the control of blood glucose (17, 18).

In this study, ADP was administrated in mice to test its impact in the blood glucose. We found that ADP exhibited an activity in the induction of blood glucose through promotion of hepatic gluconeogenesis. The mechanism might involve in the induction of NADH and activation of the P2Y13 pathway in hepatocytes. ATP was examined in the liver, muscle, kidney colon and plasma of DIO mice using metabolomics technology. But no elevation was observed in ATP in those tissues and plasma of DIO mice.

## Materials and Methods

### Animals and Reagents

Male C57BL/6 mice (6 weeks old, SPF grade) were purchased from the Shanghai Xipuer Bikai Laboratory Animal Co. Ltd. (Shanghai, China). The mice were maintained in the animal facility (SPF grade) of Shanghai Jiao Tong University. The facility had a control in temperature (22 ± 2°C) and humidity (60 ± 5%), and 12h dark/light cycle. The control group (n=24) was fed the Chow diet (13.5% kcal from fat, 60% kcal from carbohydrate, 26.5% kcal from protein, Shanghai Slack Laboratory Animal Co. Ltd.). The obese mouse model (n=20) was made by feeding the mice on a high fat diet (HFD, D12492, 60% kcal from fat, 20% kcal from carbohydrate, 20% kcal from protein, Research Diets, USA) for 16 weeks. All procedures were conducted in accordance with the animal protocol approved by the Institutional Animal Care and Use Committee (IACUC) of the Shanghai Sixth People’s Hospital affiliated to the Shanghai Jiao Tong University. ATP (987-65-5, Lablead Biotech, Beijing), ADP (A2754, Sigma, USA), AMP (4578-31-8, Lablead Biotech, Beijing) were injected intraperitoneally in mice after overnight fasting at dosages as indicated in each experiment.

In the intraperitoneal injection, the solutions for ADP, ATP and AMP were all prepared in saline. In the cell culture study, the ADP solution was prepared in the phosphate buffered solution (PBS). MRS2211 (sc204101, Santa Cruz, USA) and MRS2179 (M3808, Sigma, USA) were prepared in PBS and used in the cell culture studies.

### Glucagon Secretion From α-Cells

Mouse pancreatic αTC1-6 cells were obtained from the American Type Culture Collection (ATCC CRL-2934, Rockville, MD, USA). Cells were maintained at 37°C and 5% CO_2_ in high glucose (25 mM) Dulbecco’s minimum essential medium (DMEM) with 10% fetal bovine serum (FBS) (10099141C, Gibco, Grand Island, USA) supplemented with 1% penicillin–streptomycin (15140122, Gibco, Grand Island, USA). αTC1-6 cells were plated in 48-well culture plates and grown to 50–70% confluence. On the day of experiment, cells were washed twice with Krebs-Ringer buffer (KRB 115mM NaCl, 24mM NaHCO_3_, 5mM KCl, 1mM MgCl_2_, 2.5mM CaCl_2_, 25mM HEPES, pH 7.4, and 0.1% BSA) and preincubated in KRB containing MRS2211 (50 μM) or MRS2179 (50 μM) for 30min. The medium was removed, and cells were incubated in fresh KRB containing ADP (2.5 mM), ADP+MRS2211, ADP+MRS2179 for 1h. The medium was removed and cells were preincubated in KRB (1 mM glucose) for 30min. Then, the cells were incubated in fresh KRB (6 mM glucose) for 1h to induce glucagon secretion. The cell cultu North Institute of Biotechnology Co. Ltd (S10950157, Beijing, China). All glucagon secretion data were normalized to the cellular glucagon content. Re supernatant was collected for detection of glucagon secretion. Glucagon in cells was extracted by acid-ethanol. Secreted glucagon and intracellular glucagon were measured using ^125^I glucagon radioimmunoassay assay kit purchased from Beijing

### Insulin Tolerance Test (ITT) and Pyruvate Tolerance Test (PTT)

ITT was conducted with intraperitoneal injection (i.p.) of human insulin (1 U/kg, HY-P0035, MedChemExpress, USA) after 4h fasting. PTT was conducted with intraperitoneal injection of sodium pyruvate (2 g/kg) in mice after 16h fasting (overnight). The blood glucose was tested in the tail vein blood using the Roche’s blood glucose meter (ACCU-CHEK, Active) with the test strips. The glucose test was conducted at 0.5, 1, 1.5, and 2h.

### Hormone Assay

The blood was collected from the mice at 1h after intraperitoneal injection of ADP. The collection was made with the heparin tubes at the retro orbital sinus after 20min anesthesia with 1% pentobarbital at a dose of 50 mg/kg. The blood volume was about 500 -1000 μL/mouse. The plasma was obtained by centrifuging the blood at 3000g and stored at -80°C before the assays. Insulin and glucagon were measured in the plasma with the insulin ELISA Kit (90080, Crystal Chem, Downers Grove, USA) and glucagon ELISA Kit (DGCGO, R&D Systems, Minneapolis, USA). For other hormones, ultra-high performance liquid chromatography-tandem mass spectrometry (UPLC-MS/MS) (ACQUITY UPLC-Xevo TQS, Waters Corp., Milford, MA, USA) was used in the detection of aldosterone, corticosterone, T3 and T4. QuanMET software (v2.0, Metabo-Profile, Shanghai, China) was used to process the raw data of UPLC-MS/MS, and to perform peak integration, calibration and quantification of each metabolite.

### qRT-PCR

mRNA of *G6pas*, *Fbp1*, *Pck1* (*Pepck1*) was quantified with qRT-PCR according to a published protocol (19). Total RNA was extracted from the liver tissues and primary hepatocyte using the RNA Extraction Reagent (R401-01, Vazyme Biotech, China), which was quantified by SpectraMax i3× (Molecular Devices). mRNA was transcribed into cDNA using the HiScript^®^ II Q RT SuperMix for qPCR (+g DNA wiper) (R223-01, Vazyme Biotech, China), which was quantified with ChamQ™ Universal SYBR qPCR Maser Mix (Q711-02, Vazyme Biotech) on Light Cycle 480II (Roche). The primer sequences were listed as follows: *G6pase* forward, 5’-GCCTTCTATGTCCTCTTTCCC-3’ and reverse, 5’-GCGTTGTCCAAACAGAATCC-3’; *Fbp1* forward, 5’-ATGGATTGTGGTGTCAACTG-3’ and reverse, 5’-CTCATTAAGGCTGTAGATGTTACC-3’; *Pck1* forward, 5’-GGAAGAACAAGGAGTGGAGAC-3’ and reverse, 5’-CAGGCAGGGTCAATAATGGG-3’; *P2Y1* forward, 5’-GACTGACTGGATCTTCGGGGA-3’ and reverse 5’-CCACCACAATGAGCCACACC-3’; *P2Y12* forward, 5’-CCACTAACTAGTATTCCCGGAGAC-3’ and reverse, 5’-GATGAGCCCAGCAAAGAACA-3’; *P2Y13* forward, 5’-GCCTTTCAAAATCCTTTCCGA-3’ and reverse, 5’-TGTTTTTGCGAAAGCCGTCT-3’; *18S* forward, 5’-GCCGCTAGAGGTGAAATTCT-3’ and reverse, 5-TCGGAACTACGACGGTATCT-3; The results were normalized as 18S mRNA level.

### Western Blot

Western blotting was conducted according to a protocol described elsewhere (19). Proteins of interest were investigated with the primary antibodies to p-AMPK (ab133448), AMPK (ab207442) from Abcam (Cambridge, UK), p-Akt (Thr-308, 13038), Akt (9272), p-IRS-1 (Ser1101, 2385S), p-IRS-1 (Ser302, 2384S), IRS-1 (2382), p-CREB (9198S) and CREB (9197S) from the Cell Signaling Technology (Boston, USA). The signal was detected with chemiluminescent detection system (E412-01-AA, Western Lightning ECL; Vazyme). The signal was collected with a CCD camera system (Light-Capture; ATTO, Tokyo, Japan) and the statistical analysis was conducted with the ImageJ program.

### Targeted Metabolomics

The fresh liver tissue was collected from the mice 1h after ADP injection (125 mg/kg, i.p.). The liver, skeletal muscle (quadriceps femoris), kidney and colon were collected from DIO mice (16 wks, HFD) under random feeding. The tissues were frozen in liquid nitrogen immediately after collection and kept in -80°C freezer for 1-2 wks until analysis. The metabolites (34 small molecule chemicals) in the energy metabolism pathways including glycolysis, TCA cycle, oxidative phosphorylation, and pentose phosphate pathways were analyzed using the ultraperformance liquid chromatography (Agilent 1290 Infinity LC) coupled with triple quadrupole mass spectrometry (5500 QTRAP, AB SCIEX). The data were analyzed with multiquant program against the standard compounds.

### Primary Hepatocytes

The primary hepatocytes were prepared using a modified two-step perfusion protocol. The mice were anesthetized with 1% pentobarbital, and a puncture was made in the inferior vena cava with an indwelling needle to perfuse the liver with the pre-perfusion fluid at 24 mL/mouse. The perfusion fluid flowed out from the hepatic portal vein. The infusion was continued with 40 mL/mouse of the 37°C post-perfusion solution containing the IV type collagenase (C5138, Sigma, USA). The perfused liver was cut with surgical scissor and then crushed in a fresh serum-free medium to obtain the hepatocyte suspension. The hepatocyte suspension was filtered with a 200-mesh screen and centrifuged at 500 r/min for 1 minute. The cell pellets were suspended in the mixture of Percoll cell separation solution, serum-free medium and 10×PBS, and centrifuged at 700 r/min for 20 minutes to obtain the primary hepatocytes in the pellet. The pellet was suspended in 10 mL of DMEM medium (containing 10% FBS and 1% penicillin-streptomycin) and inoculated in the precoated six-well plate containing 0.1% collagen at a density of 1×10^6^/mL. Each well contained 2 mL of the complete medium and the cells were cultured in a 37°C, 5% CO_2_ incubator for 6h until complete attachment to the wall. The cells were cultured in fresh culture medium for additional 12h before the ADP treatment.

### Detection of Liver Glycogen Content

The liver glycogen was determined with the anthrone method. Glycogen was extracted with the alkaline solution, and the glycogen content was determined by anthrone color reagent under strong acid conditions with Micro Glucogen Content Assay Kit (BC0345, Solarbio, Beijing, China) according to the manufacturer’s instruction.

### Statistical Analysis

All *in vitro* experiments were conducted at least three times with consistent results. The protein signals were quantified in the immunoblots using the ImageJ software. The data of ITT and PTT were statistically analyzed with the two-way ANOVA, and other *in vivo* data were statistically analyzed with the one-way ANOVA. The *in vitro* data were analyzed with Student T-test. All statistical analyses were performed using SPSS with a statistical significance at p < 0.05.

## Results

### ADP Increased Blood Glucose in Normal Mice

**Figure 1:**
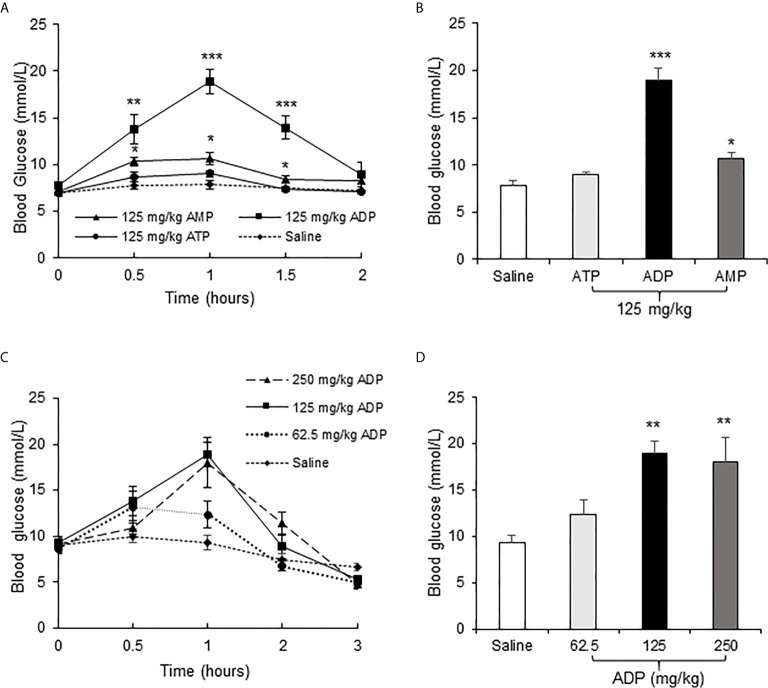
ADP increased blood glucose in mice. **(A)** Comparison of the effects of ATP, ADP and AMP on induction of blood glucose. Blood glucose was determined in the tail vein blood at multiple time points (0, 0.5, 1, 1.5, 2h) after intraperitoneal (i.p.) injection of ATP, ADP, AMP (125 mg/kg, n=6). **(B)** Blood glucose at 1h after ATP, ADP, AMP injection. The data is derived from the figure **(A)**. **(C)** Dose effect of ADP. The blood glucose was determined at multiple time points (0, 0.5, 1, 2, 3h) at three dosages (62.5, 125, 250 mg/kg) of ADP, n=6). **(D)** Blood glucose at 1h after ADP injection. The data was derived from the figure **(C)**. The data in this figure represents mean ± SEM. *p < 0.05; **p < 0.01; ***p < 0.001 vs. the saline control group.

To test the effect of ATP in insulin resistance, a bolus injection of ADP was administrated intraperitoneally into the overnight fasted mice, at a dosage of 125 mg/kg body weight, to promote mitochondria ATP synthesis. In the control mice, normal saline (NS) was injected into the mice at the same volume. ATP and AMP were used at the same dosages in additional two groups of mice, respectively, to test the unique activity of ADP. Blood glucose was monitored in the tail vein blood of mice within 2h of ADP injection at an interval of 0.5h. The blood glucose was increased significantly in the ADP group (**Figures 1A, B**). The increases were detected at three time points (0.5, 1 and 1.5h) with a peak at 1h (**Figure 1B**). AMP also exhibited a significant activity, but it was much lower than that of ADP (**Figures 1A, B**). ATP did not show a significant activity (**Figures 1A, B**). To characterize the ADP activity further, a dose-dependent study was conducted at three dosages (62.5, 125, 250 mg/kg) of ADP. Compared to that of 125 mg/kg, the higher dosage (250 mg/kg) did not exhibit an extra activity, and the lower dosage (62.5 mg/kg) exhibited less activity (**Figures 1C, D**). These observations suggest that ADP was the major purine product in the induction of blood glucose. The most effective dosage of ADP was 125 mg/kg.

### ADP Increased Hepatic Gluconeogenesis

**Figure 2:**
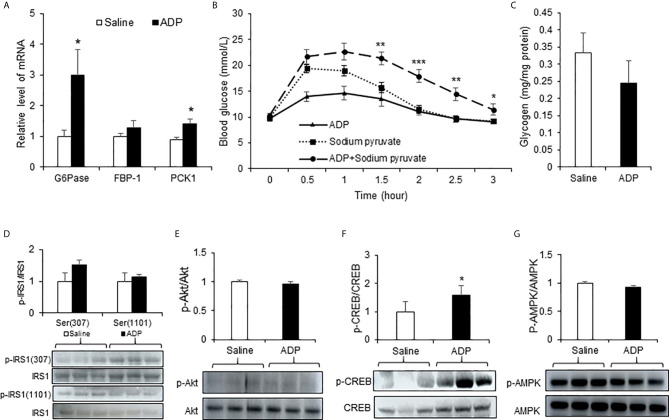
ADP induced hepatic gluconeogenesis in mice. **(A)** Expression of gluconeogenic genes. The mRNA levels of gluconeogenic genes (*G6pase*, *Fbp1*, *Pck1*) were determined with qRT-PCR in the liver tissue of mice after injection of ADP (125 mg/kg) at 1h (n=6). **(B)** Pyruvate tolerance test (PTT). The mice were injected i.p. with sodium pyruvate (2 g/kg) and ADP (125 mg/kg) after 16h fasting (n=9). **(C)** Liver glycogen. Glycogen abundance was determined in the liver tissue of mice after ADP (125 mg/kg) injection at 1h (n=6). *p < 0.05; **p < 0.01; ***p < 0.001 vs. the sodium pyruvate group. Phosphorylation level of IRS1 **(D)**, Akt **(E)**, CREB **(F) **, AMPK **(G)** in the liver tissue at 1h after ADP injection (n=3). *p < 0.05 vs normal saline control group. The data in this figure represents mean ± SEM.

Liver is the primary organ in glucose production under the fasting conditions. In search for the target organ of ADP, we examined liver responses in gene expression, pyruvate tolerance and glycogen content. The test was conducted at the peak time of ADP activity at 1h of injection. An increase was observed in *G6pase* and *Pck1* mRNA (**Figure 2A**). The pyruvate tolerance test was conducted to test the hepatic gluconeogenesis, in which the pyruvate injection increases blood glucose. ADP significantly promoted the pyruvate effect with additional elevation of the blood glucose (**Figure 2B**). Glycogen content was examined in the liver for glycogenolysis. A trend of reduction was observed in the ADP-treated group although the change was not significant (**Figure 2C**). These results support that ADP targeted liver in the induction of blood glucose through promotion of hepatic gluconeogenesis.

The hepatic gluconeogenesis is regulated by insulin and glucagon. The insulin and glucagon signaling pathways were examined in the liver tissue to understand the ADP activity. Serine phosphorylation of IRS-1 and Akt was examined in the insulin signaling pathway. No significant change was observed in the ADP-treated group (**Figures 2D, E**). Phosphorylation of CREB (p-CREB) was determined in the glucagon signaling pathway, and an increased activity was observed in the ADP group (**Figure 2F**). AMPK was examined in the same condition and no change was observed in the ADP group (**Figure 2G**). These data suggests that ADP may promote gluconeogenesis through the impact in the glucagon, but not the insulin signaling pathway.

### ADP Induced NADH and cAMP Levels in Liver

**Figure 3:**
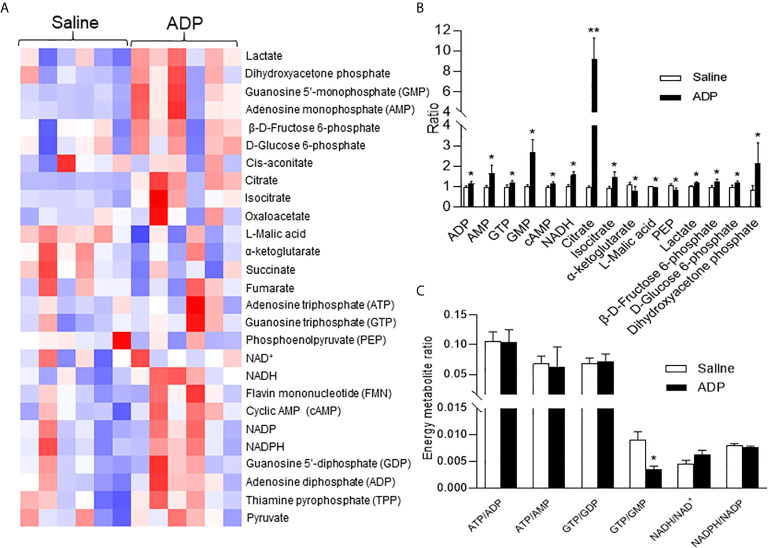
ADP induced NADH in promotion of gluconeogenesis. Metabolite profile was determined in the liver using metabolomics technology at 1h after ADP (125 mg/kg) administration. **(A)** Heat map of liver metabolites after ADP injection. **(B)** Substrate ratio. **(C)** Energy currency and reduction equivalent ratio. The data in the bar figures represent mean ± SEM (n=6). *p < 0.05; **p < 0.01 vs. the saline control group.

The hepatic gluconeogenesis is influenced by metabolites of glucose and other substrates. To understand the ADP activity, a targeted metabolomics approach was employed to monitor change in the energy-related metabolites in the liver tissue. Thirty-four metabolites including ATP and NADH (nicotinamide adenine dinucleotide) were examined at 1h of ADP injection. In the metabolites, ATP was not increased, but other purine derivatives including ADP, AMP, GTP, and GMP were all elevated (**Figures 3A, B**). Additionally, the NADH and cAMP (cyclic AMP) levels were increased in the ADP-treated group (**Figure 3B**). The glucose-related intermediate metabolites (phosphoenolpyruvate, β-d-fructose 6-phosphate, d-glucose 6-phosphate, dihydroxyacetone phosphate and lactic acid) were all increased (**Figure 3B**). In the tricarboxylic acid cycle, citrate and isocitrate were increased at the initial steps, while α-ketoglutarate and malic acid were decreased at the intermediate steps (**Figure 3B**). There was a reduction in the GTP/GMP ratio, but no change in the ratio of ATP/ADP, ATP/AMP, GTP/GDP, etc. (**Figure 3C**). These results suggest that the intermediate metabolites for gluconeogenesis were increased in the liver by ADP. The elevation in ADP, NADH and cAMP is more interesting in the induction of gluconeogenesis in the ADP-treated group.

### ADP Activated the cAMP/PKA/CREB Pathway Through Purinergic Receptor P2Y13

**Figure 4:**
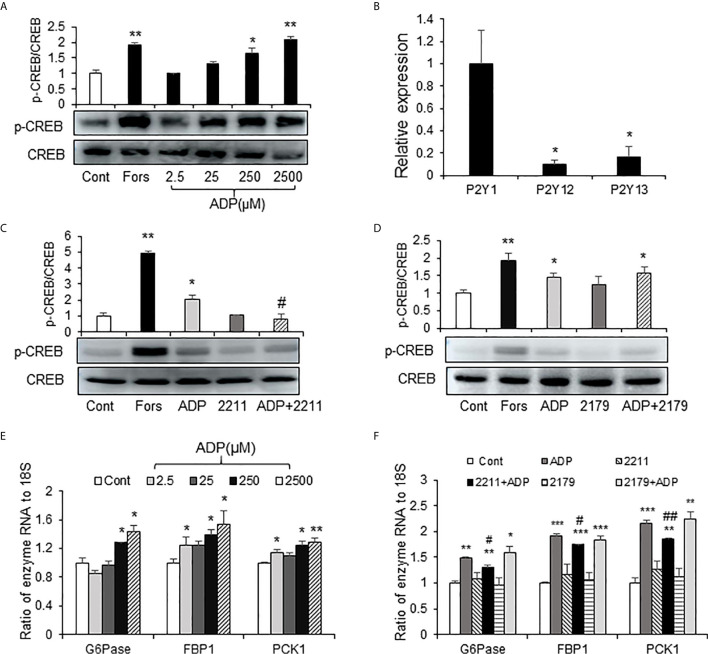
ADP activated the cAMP/PKA/CREB pathway through the P2Y13 receptor in primary hepatocytes. **(A)** Induction of CREB phosphorylation by ADP. The p-CREB and CREB signals were determined in the primary hepatocyte after ADP (2.5, 25, 250, 2500 μM) treatment. Forskolin (50μM) was used as a positive control. The primary hepatocytes were incubated with 2.5 mM ADP for 1h. **(B)** Relative expression of ADP receptors of P2Y1, P2Y12 and P2Y13 in the mouse liver (n=3). *p < 0.05 vs. the P2Y1 relative expression. **(C)** Blockage of P2Y13. The receptor was blocked with the chemical antagonist MRS2211 (50 μM). **(D)** Blockage of P2Y1. The receptor was blocked with the chemical antagonist MRS2179 (50 μM). The antagonists were added 30min before ADP treatment. The experiments were repeated at least three times with consistent results. Representative blots are presented. **(E)** Induction of gluconeogenic genes by ADP. The mRNA levels of gluconeogenic genes (*G6pase*, *Fbp1*, *Pck1*) were determined with qRT-PCR after ADP (2.5, 25, 250, 2500 μM) treatment for 1h. **(F)** The effects of MRS2211(50 μM) and MRS2179 (50 μM) on the mRNA expression was determined. The data in bar figure represents mean ± SEM. *p < 0.05; **p < 0.01; ***p < 0.001 vs. the control group; ^#^p < 0.01; ^##^p < 0.01 vs. the ADP-stimulated cultures.

The elevation in cAMP and p-CREB suggest that the glucagon signaling pathway is activated in the liver of ADP-treated mice. However, it was unknown if this was a direct effect of ADP on the hepatocytes. To address the issue, an *in vitro* study was carried out using the mouse primary hepatocytes in the cell culture, and the activity of cAMP/PKA pathway was examined following ADP treatment of the cells. The pathway was activated by ADP as indicated by an increase in the p-CREB signal (**Figure 4A**), a kinase substrate of PKA (protein kinase A). ADP was tested at four dosages (2.5, 25, 250 and 2500 μM) in the study and the activation was observed at three dosages except 2.5 μM (**Figure 4A**). The p-CREB signal was significantly enhanced at 250 μM, and the strongest activity was observed at 2500 μM. Forskolin (50 μm) was used in the positive control (**Figure 4A**). The cAMP/PKA pathway was associated with the G protein-coupled receptors in most cases. ADP has three G protein-coupled receptors, P2Y1, P2Y13, and P2Y12 (20). In order to detect the receptor expression in the liver, we tested mRNA of these receptors, and found that P2Y1 mRNA was the highest, while the expression of P2Y13 and P2Y12 was less (**Figure 4B**). The receptors were tested in the mechanism of ADP activity with the specific chemical blockers. The blocker for P2Y13 receptor (MRS2211) was effective in the suppression of the ADP activity (**Figure 4C**). The blocker for P2Y1 receptor (MRS2179) did not exhibit the same activity (**Figure 4D**). ADP induced mRNA expression of key enzymes of gluconeogenesis and the activity was inhibited by the P2Y13 receptor blocker (MRS2211) in the cellular model (**Figures 4E, F**). The P2Y12 receptor was not tested in this study. The observations suggest that ADP interacted with the P2Y13 receptor in the surface of hepatocytes to activate the cAMP/PKA signaling pathway in the induction of hepatic gluconeogenesis.

### ADP Induced Glucogenic Hormones in Blood

**Figure 5:**
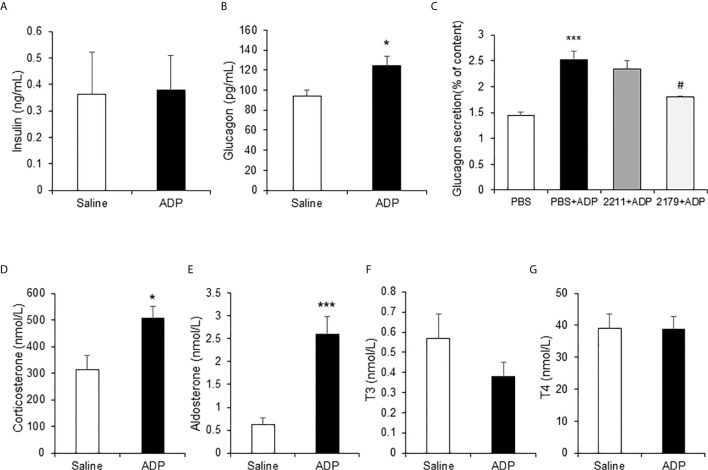
ADP induced glucogenic hormones. The hormones of insulin, glucagon, corticosterone, aldosterone and thyroid hormones were examined in the mouse plasma after ADP injection at 1h. The levels of insulin and glucagon in plasma were detected with ELISA (n=4). **(A)** Insulin; **(B)** Glucagon. **(C)** ADP induction of glucagon secretion. αTC1-6 cells were treated with 2.5 mM ADP and pretreated with MRS2211 (50 μM) or MRS2179 (50 μM) for 0.5h in the study. The cellular experiments were repeated at least three times with consistent results. The hormones were determined in the plasma after ADP injection for 1h (n=6). **(D)** Corticosterone; **(E)** Aldosterone; **(F)** T3; **(G)** T4. The data in the bar figure represents mean ± SEM. *p < 0.05; ***p < 0.001 vs. the control group in **(B, D, E)**. ***p < 0.001 vs. the PBS group; ^#^p < 0.05 vs. the PBS+ADP group in the panel **(C)**.

In the mice, ADP may act through an impact on the hormone levels for an indirect action. To test the possibility, the gluconeogenesis-related hormones were examined in the plasma of ADP-treated mice at 1h of ADP injection. The hormones were quantified with ELISA and mass spectrometry, respectively. The gluconeogenic hormones including glucagon and corticosterone were increased in the plasma of ADP-treated group together with aldosterone, but other hormones including insulin and thyroid hormones (T3 and T4) remained unchanged (**Figure 5**). Glucagon was secreted by the pancreatic α-cells, corticosterone and aldosterone by the adrenal gland. To test ADP impact in glucagon secretion, we treated the α-cell line with ADP in the cell culture. ADP induced glucagon secretion from the cells leading to hormone elevation in the supernatant. MRS2179 can block this effect, but MRS2211 cannot (**Figure 5C**). The data suggest that ADP might act in the pancreas and adrenal gland to regulate the hormone secretion for the hormone elevation in the blood, which represents an indirect mechanism for ADP action in the induction of blood glucose.

### ADP Intensified Insulin Resistance in DIO Mice

**Figure 6:**
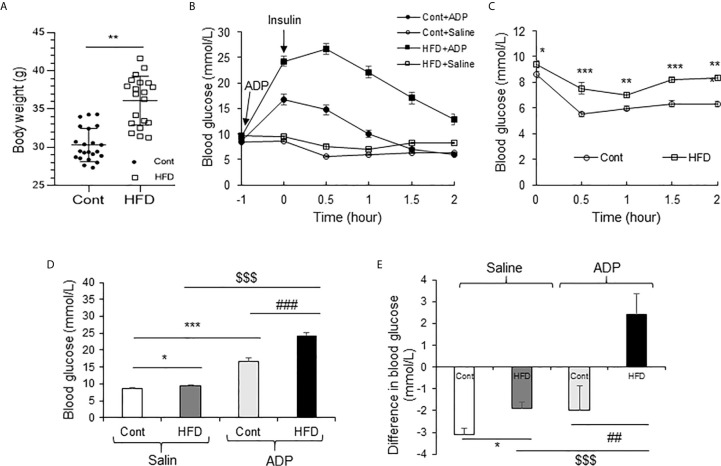
ADP impaired insulin sensitivity in the DIO mice. **(A)** Body weight of Chow diet mice and high-fat diet mice (19-week-old, n=20). **(B)** Insulin tolerance test (ITT) after ADP injection. Normal mice and DIO mice were injected with ADP (125 mg/kg) or saline 1 hour before i.p. injecting insulin (1 U/kg). **(C)** ITT of normal mice and DIO mice without ADP. The data was derived from panel A.** (D)** Blood glucose after ADP injection at 0.5 h from panel A.** (E)** Blood glucose before and after insulin (1 U/kg) injection at 0.5 h. The data in this figure represent mean ± SEM (n=10). *p < 0.05; **p < 0.01; ***p < 0.001 vs. control group; ^##^p < 0.01; ^###^P < 0.001 vs. the ADP control group; ^$$$^P < 0.001 vs. the normal HFD group.

Above observations were made in the lean mice and the results suggested a transient insulin resistance in the liver from the ADP treatment. The ADP activity might deteriorate insulin resistance in the pathological condition. To test the possibility, insulin resistance was examined in DIO mice through the insulin tolerance test (ITT). The DIO mice were pretreated with ADP for 1h before ITT. The lean mice were used in the control. The body weight data of DIO mice and lean mice are presented in **Figure 6A**. ITT was impaired in the lean mice by ADP for the elevated blood glucose (**Figure 6B**). The DIO mice exhibited impaired insulin tolerance relative to the lean mice (**Figure 6C**). The impairment was intensified by ADP for an extra increase in the blood glucose (**Figures 6B, C**). The insulin action was significantly attenuated in the ADP group as indicated by the delayed glucose reduction upon the insulin injection. The glucose elevation continued for additional 0.5h after the insulin injection in the ADP group. The data suggest that the insulin resistance became worse in the DIO mice after ADP treatment. To illustrate the insulin resistance, the blood glucose levels were plotted at the time 0.5h post insulin injection (**Figure 6D**), and magnitude of the insulin-induced reduction in glucose was plotted at the same time for the insulin action (**Figure 6E**). The results suggest that ADP intensified insulin resistance in the DIO mice.

### NADH Is a Marker for Hepatic Gluconeogenesis in DIO Mice

**Figure 7:**
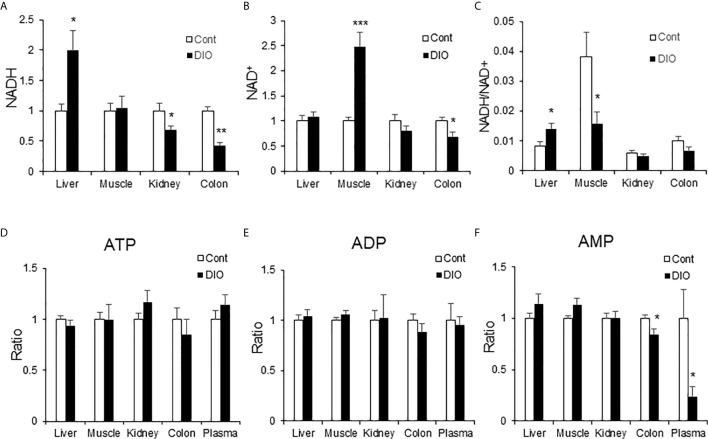
NADH was elevated in the liver of DIO mice. The energy-related metabolites were quantified by the targeted metabolomics in multiple tissues (liver, muscle, kidney, colon and plasma) of DIO mice at 16 wk on HFD. The control mice were on chow diet. The relative abundance of NADH, NAD+, ATP, ADP and AMP are presented in the bar figures. **(A)** NADH; **(B)** NAD+; **(C)** Ratio of NADH/NAD+; **(D)** ATP; **(E)** ADP; **(F)** AMP. The data represents mean ± SEM (n=6). *p < 0.05; **p < 0.01; ***p < 0.001 vs. the control group.

NADH is a substrate of the respiratory chain to support ATP production in mitochondria. NAD^+^ is the product of NADH following loss of “H^+^” in the respiration-mediated oxidation. As an indicator of energy surplus, NADH/NAD^+^ ratio was elevated in the liver of DIO mice for hepatic insulin resistance (21). However, the ratio remained to be tested in other glucogenic organs, such as kidney and colon. To address the issue, NADH and NAD^+^ were quantified in the liver, kidney and colon of DIO mice using the targeted metabolomics approach for the energy-related metabolites. NADH was significantly elevated in the liver, but not in the kidney and colon (**Figure 7A**). NADH was even decreased in the kidney and colon. NAD^+^ was not altered in the liver and kidney, but significantly elevated in the muscle and decreased in the colon (**Figure 7B**). The NADH/NAD^+^ ratio was elevated in the liver but decreased in the muscle (**Figure 7C**). No change was found in the kidney and colon. In the assay, no change was observed for ATP, ADP and AMP in the tissues except a reduction of AMP in the colon and plasma (**Figures 7D–F**). These data suggest that NADH and the NADH/NAD^+^ ratio might be specific metabolic markers for gluconeogenesis in the liver of DIO mice. The parameter might not apply to other gluconeogenic organs.

## Discussion

Current study demonstrates a new activity of ADP in the induction of hepatic gluconeogenesis. This activity is supported by the phenotype of global CD39-KO mice for insulin resistance and hyperinsulinemia (22). In the extracellular matrix, ADP is derived from ATP hydrolysis, and cleared by an ectoenzyme (CD39) (13). In the CD39-KO mice, ADP elevation is expected from the deficient clearance of ADP due to CD39 gene inactivation. However, the ADP role remains to be proved in the liver of CD39-KO mice. In current study, ADP was found to intensify the hepatic insulin resistance in the DIO mice. ADP promoted hepatic gluconeogenesis by impairing the pyruvate tolerance in the lean mice. The signaling pathway of ADP was explored with a focus on the purinergic receptors in the liver. ADP was found to activate the G-protein coupled receptor P2Y13 in the primary hepatocyte culture. According to the receptor activity identified in other tissues, ADP might generate two different effects on the cAMP/PKA/CREB pathway through an interaction with the P2Y13 receptor. At the μmol/L level, ADP activates the pathway (23, 24). At the nano-molar level, ADP suppresses the pathway (23). However, the P2Y13 pathway has not been reported in the liver. Current study provides data to reveal the pathway in hepatocytes. In a pharmacological study, activation of the P2Y13 receptor by a chemical ligand, diadenosine tetraphosphate (Ap4A), elevates the blood glucose in mice (25). However, the target organ of Ap4A has been unknown for the effect. Current study demonstrates that the liver is an ADP target organ. In a published study of P2Y13 knockout mice (26), the gene expression profile in liver suggests a reduction in the cAMP/PKA/CREB pathway activity. The expression of genes in the fatty acid oxidation pathway was decreased and expression of genes in the biosynthesis pathway for cholesterol and fatty acids was increased. Current study suggests that liver is a target organ of ADP in the induction of blood glucose, and the signaling activity involves a pathway of P2Y13/cAMP/PKA/CREB. However, the role of P2Y12 remains to be tested in the ADP activity.

Our data reveal an indirect mechanism for the ADP activity in the induction of liver glucose production. The hormones (glucagon and corticosterone) were induced by ADP in the blood of lean mice, which was found by screening of the hormones in the plasma of ADP-treated mice. In the cell culture, ADP induced glucagon secretion in α-cells, suggesting that ADP may target the pancreas in the induction of glucose. The adrenal gland might be another target organ of ADP in addition to the pancreas and liver. Hyperglucagonemia is a risk of hyperglycemia in the type 2 diabetes (27). However, the mechanism remains elusive for the hyperglucagonemia. ADP may represent a potential mechanism as the level may increase in the pancreatic islet under insulin hypersecretion. The pancreatic β-cells secrete ATP together with insulin in the insulin granule (14, 15). The extracellular ATP has been documented in the promotion or inhibition of insulin secretion in a feedback manner in the islet (18, 28). However, the role of extracellular ADP remains largely unknown. Extracellular ATP increases the ADP level in the extracellular matrix after hydrolysis. In current study, ADP was demonstrated to induce glucagon secretion in the pancreatic α-cells. Additionally, ADP might regulate corticosterone secretion in the adrenal gland. These gluconeogenic hormones may mediate the ADP activity in the induction of hepatic gluconeogenesis as an indirect mechanism. ADP was reported to inhibit insulin secretion in a β-cell line and the activity was blocked by the P2Y13 receptor blocker (MRS2211) (29). The ADP effect on insulin was not observed in current study, which might be related to a difference in the ADP dosage or observation window. Induction of glucagon and corticosterone by ADP represents a novel mechanism for the induction of blood glucose by ADP, which has not been reported in the literature yet (18, 30).

This study provides evidence that the intracellular NADH, but not ATP, may mediate the ADP signal in the hepatic gluconeogenesis. Both NADH and ATP were reported as the energy surplus signals for insulin resistance. However, the two molecules have not been compared carefully in a study. Energy surplus is a major risk factor for insulin resistance. The identity of surplus signal remains controversial. Intracellular ATP was proposed as a signal for insulin resistance in models including the mice and human, in which ATP was quantified using the luciferase-based assay (19, 31–34). In this study, a more advanced technology of the ultraperformance liquid chromatography coupled with triple quadrupole mass spectrometry was used to quantify ATP and NADH in the tissues. No elevation was observed in ATP in the liver of DIO mice or ADP-treated lean mice. In contrast, the elevation in NADH was observed in the liver of both models. NADH is mainly produced in the TCA cycle, glycolysis and β-oxidation to carry electron to the mitochondrial respiration chain. NADH has recently been found as a metabolic trait for hepatic insulin resistance in both human and mice (21). However, the trait has not been tested in other gluconeogenic organs except the liver. NADH was quantified in three glucogenic tissues together with the skeletal muscle in current study. The elevation was observed only in the liver, but not in the kidney, colon and skeletal muscle in the DIO mice. The elevation was also observed in the liver of ADP-treated lean mice. These observations suggest that NADH is a metabolic marker for hepatic gluconeogenesis in the mechanism of insulin resistance. In the skeletal muscle, NAD^+^ was increased leading to a reduction of NADH/NAD^+^ ratio in the DIO mice.

In summary, current study suggests that ADP may induce the blood glucose through direct and indirect mechanisms. The direct mechanism may involve activation of the P2Y13/cAMP/PKA/CREB signaling pathway in hepatocytes by ADP and the indirect mechanism may involve induction of the gluconeogenic hormones by ADP. These mechanisms may lead to the induction of the hepatic gluconeogenic activity. NADH was elevated in the liver of DIO mice and ADP-treated mice, which was coupled with elevated gluconeogenesis. ATP was not elevated in the livers of DIO mice and ADP-treated mice. We conclude that NADH is a marker molecule for hepatic gluconeogenesis in the mechanism of insulin resistance. The NADH activity may not apply to other gluconeogenic organs and skeletal muscle in the pathogenesis of insulin resistance.

## Data Availability Statement

The raw data supporting the conclusions of this article will be made available by the authors, without undue reservation.

## Ethics Statement

The animal study was reviewed and approved by Institutional Animal Care and Use Committee (IACUC) of the Shanghai Sixth People’s Hospital affiliated to the Shanghai Jiao Tong University.

## Author Contributions

XC, XY, SZ, LW, YX, SP, YZ, YP, JL, and XZ conducted the experiments, analysed the data, and prepared the figures and manuscript draft. XH, W-yH, WJ, and JY came up with the concept, designed the study, interpreted the data and drafted the manuscript. All authors contributed to the article and approved the submitted version.

## Funding

The project was supported by the National Key R&D Program of China (2018YFA0800603) and a project (19ZR1439000) of the Shanghai Association for Science and Technology to JY.

## Conflict of Interest

The authors declare that the research was conducted in the absence of any commercial or financial relationships that could be construed as a potential conflict of interest.
